# The DNA glycosylase NEIL2 plays a vital role in combating SARS-CoV-2 infection

**DOI:** 10.21203/rs.3.rs-1690354/v1

**Published:** 2022-05-27

**Authors:** Tapas Hazra, Nisha Tapryal, Anirban Chakraborty, Kempaiah Rayavara, Maki Wakamiya, Azharul Islam, Lang Pan, Jason Hsu, Vivian Tat, Junki Maruyama, Koa Hosoki, Ibrahim Sayed, Joshua Alcantara, Vanessa Castillo, Courtney Tindle, Altaf Sarker, Victor Cardenas, Gulshan Sharma, Laura Crotty Alexander, Sanjiv Sur, Gourisankar Ghosh, Slobodan Paessler, Debashis Sahoo, Pradipta Ghosh, Soumita Das, Istvan Boldogh, Chien-Te Tseng

**Affiliations:** The University of Texas Medical Branch at Galveston; The University of Texas Medical Branch at Galveston; The University of Texas Medical Branch at Galveston; The University of Texas Medical Branch at Galveston; The University of Texas Medical Branch at Galveston; The University of Texas Medical Branch at Galveston; The University of Texas Medical Branch at Galveston; The University of Texas Medical Branch; University of Texas Medical Branch; University of Texas Medical Branch; Baylor College of Medicine; University of California, San Diego; UC San Diego Health; University of California San Diego; University of California, San Diego; Lawrence Berkeley National Laboratory; The University of Texas Medical Branch at Galveston; The University of Texas Medical Branch at Galveston; University of California; Baylor College of Medicine; University of California, San Diego; University of Texas Medical Branch at Galveston; University of California, San Diego; University of California San Diego; UCSD; University of Texas Medical Branch at Galveston; University of Texas Medical Branch

## Abstract

Compromised DNA repair capacity of individuals could play a critical role in the severity of SARS-CoV-2 infection-induced COVID-19. We therefore analyzed the expression of DNA repair genes in publicly available transcriptomic datasets of COVID-19 patients and found that the level of NEIL2, an oxidized base specific mammalian DNA glycosylase, is particularly low in the lungs of COVID-19 patients displaying severe symptoms. Downregulation of pulmonary NEIL2 in CoV-2-permissive animals and postmortem COVID-19 patients validated these results. To investigate the potential roles of NEIL2 in CoV-2 pathogenesis, we infected *Neil2*-null (*Neil2^−/−^*) mice with a mouse-adapted CoV-2 strain and found that *Neil2^−/−^* mice suffered more severe viral infection concomitant with increased expression of proinflammatory genes, which resulted in an enhanced mortality rate of 80%, up from 20% for the age matched *Neil2^+/+^* cohorts. We also found that infected animals accumulated a significant amount of damage in their lung DNA. Surprisingly, recombinant NEIL2 delivered into permissive A549-ACE2 cells significantly decreased viral replication. Toward better understanding the mechanistic basis of how NEIL2 plays such a protective role against CoV-2 infection, we determined that NEIL2 specifically binds to the 5’-UTR of SARS-CoV-2 genomic RNA and blocks protein synthesis. Together, our data suggest that NEIL2 plays a previously unidentified role in regulating CoV-2-induced pathogenesis, via inhibiting viral replication and preventing exacerbated proinflammatory responses, and also via its well-established role of repairing host genome damage.

## Introduction

Coronavirus disease-2019 (COVID-19), caused by the novel severe acute respiratory syndrome coronavirus-2 (SARS-CoV-2), remains a priority of public health concern worldwide. Like other highly pathogenic coronaviruses (CoVs), i.e., SARS-CoV and Middle East Respiratory Syndrome (MERS)-CoV, the pathogenesis of severe COVID-19 is largely attributed to diffused alveolar damage that eventually leads to the onsets of acute respiratory distress syndrome (ARDS), acute lung injury, and multiorgan failure (1-6). Recent evidences suggest that the severity of COVID-19 correlates well with a dysregulated and often exacerbated proinflammatory response, also termed a “cytokine storm” (7-10). Thus, several biologic interventions specifically targeting inflammatory cytokines and related signaling pathways are being clinically evaluated, including IL-6 inhibitors, IL-1 inhibitors, anti-TNF-α agents, corticosteroids, intravenous immunoglobulin (IVIG), and colchicine (11-16). While the efficacy and safety of these anti-inflammatory agents in COVID-19 patients are still under investigation (17), some exhibited adverse side effects in patients (10, 18-20) and could not provide a foreseeable clinical benefit. Hence, it is imperative to better understand the molecular basis of CoV-2 pathogenesis for identifying novel targets for effective interventions against COVID-19.

Recently, several studies have indicated that cytokine storm and oxidative stress contribute to the severe outcome of COVID-19 patients (21-25). Such hyperinflammation and oxidative stress can generate an excessive level of reactive oxygen species (ROS), which consequently cause damage to various cellular macromolecules, including oxidative genome damage that is primarily repaired via the base excision repair (BER) pathway (26-28). Various proteins, involved in the BER pathway, such as Poly [ADP-ribose] polymerase 1 (PARP1), 8-Oxoguanine glycosylase (OGG1) and DNA polymerase beta (POLB) have been implicated in viral pathogenesis (29-31). Additionally, several laboratories, including ours, have reported non-canonical roles of BER/single strand break repair (SSBR) proteins, including PARP1, OGG1 and Nei Like DNA Glycosylase 2 (NEIL2) in modulating innate immune response (31-36). However, the role of BER/SSBR proteins in the pathogenesis of SARS-CoV-2 remains unexplored to date. While analyzing the expression level of DNA BER/SSBR proteins, in publicly available transcriptomic databases of CoV-2 infected patients, we observed that the expression level of NEIL2, an oxidized base-specific DNA glycosylase, is significantly lower within the lungs of patients suffered from severe COVID-19, compared to those of uninfected individuals or even with those patients with milder COVID-19 symptoms. Here, we thus investigated the potential role of NEIL2 on SARS-CoV-2 infection *in vitro* and *in vivo* and explored the levels of NEIL2 expression in patients with differing severity of COVID-19. We found that the level of NEIL2 expression was inversely correlated with disease severity in patients with COVID-19. More surprisingly, 80% of *Neil2*-null mice (*Neil2^−/−^*), compared to only 20% of wild type (*Neil2^+/+^*) mice succumbed to mouse adapted SARS-CoV-2 infection. While investigating the biochemical basis, we found that NEIL2 interacts with the 5’-UTR of viral RNA, thereby blocking viral protein synthesis and eventually leading to decreased pathogenesis of SARS-CoV-2 infection in cultured cells. Together, these data strongly suggest a previously unidentified role of NEIL2 in regulating the pathogenesis of SARS-CoV-2 infection.

## Results

### The low-level expression of NEIL2 in COVID-19 patients correlates with the severity of disease

**Figure 1:**
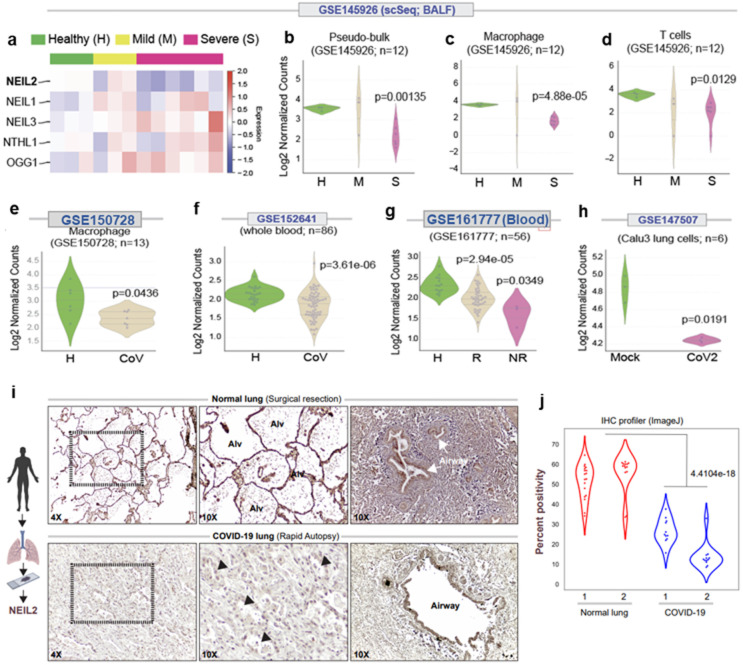
Downregulation of NEIL2 carries a poor prognosis in COVID-19 **a**) The heatmap displays changes in the expression of BER-associated DNA glycosylases in bronchoalveolar lavage samples (GSE145926) obtained from healthy controls and patients with COVID-19. **b-d**) Swarm plots display the levels of expression of NEIL2 in Pseudo-bulk (b), macrophages (c) and T-cells (d) as analyzed in healthy (H), mild (M) and severe (S) patients in the same cohort as in A. **e, f**) Swarm plots show expression of NEIL2 transcript in macrophages (GSE150728) (e) and whole blood (GSE152641) (f) from healthy controls (H) and COVID-19 patients (CoV). **g**) Swarm plots shows expression of NEIL2 in whole blood (GSE161777) from healthy controls (H) and recovered (R) or not recovered (NR) COVID-19 patients. **h**) NEIL2 expression in mock or SARS-CoV-2 infected Calu3 cells (GSE147507). **i**) Normal lung tissue obtained during surgical resection (top) or lung tissue obtained during autopsy studies of COVID-19 patients (bottom) were stained for NEIL2. Arrowheads = injured alveoli. Alv, alveolar spaces. **j**) Violin plots display the intensity of staining between healthy lung (normal) vs. SARS-CoV-2 infected (COVID-19) lung, as determined by immunohistochemistry (IHC) profiler.

SARS-CoV-2 infection-induced expression of soluble inflammatory mediators increase influx of inflammatory cells (macrophages, T cells, and, occasionally, neutrophils) to the site of infection, leading to uncontrolled inflammation, pulmonary endothelial leakage, and impairing lung function (7,8,37). CoV-2 infection and host inflammatory responses also generate ROS that are not only signal transducers but are also inducers of host genome damage, thereby triggering a DNA damage response (37). However, in the absence of any report on the mechanistic link between CoV-2 infection and host genome repair, we analyzed RNA-seq data obtained from the bronchoalveolar lavage fluids (BALFs) of the individuals suffering from severe and mild COVID-19, along with healthy individuals as controls that are available in public database (GSE 145926). Surprisingly, the level of NEIL2, among other DNA BER/SSBR proteins, was found to be significantly lower in severe COVID-19 patients relative to that of the control population (Fig. 1a-d and **Supplementary Fig. 1a, b**). Such striking findings of the transcriptomic profile in the BALF specimen were validated in three other independent datasets with whole blood transcriptomics (GSE150728, GSE152641 and GSE161777). It was determined that downregulation of NEIL2 took place primarily in the monocyte/macrophage lineages (Fig. 1e-g and **Supplementary Fig. 1c**). Importantly, downregulation of NEIL2 correlated well with disease severity, including the patients that failed to recover from COVID-19 (Fig. 1a-d and 1g). Comparative expression profiling of uninfected vs. SARS-CoV-2 infected lung epithelial cells (Calu3, GSE147507) also displayed a significant decrease in NEIL2 transcript levels post infection (Fig. 1h). Consistent with decreased levels of transcripts, a significant reduction in NEIL2 protein was also observed in the SARS-CoV-2-infected lungs, particularly in alveolar epithelial cells, compared to healthy controls (Fig. 1i,j) as analyzed by Immunohistochemical (IHC) analysis of paraffin-embedded lung specimens of COVID-19 patients.

We next investigated the expression of NEIL2 at mRNA and protein levels in SARS-CoV-2-permissive golden Syrian hamsters (38). Lung specimens were harvested at 5 days post infection (dpi) with SARS-CoV-2 (1 × 10^6^ TCID_50_), when weight loss in the animals reached its peak, for the subsequent assessment of the expression of DNA glycosylases. We found that the expression of NEIL2 protein (**Supplementary Fig. 2a,b**) was significantly low in infected lungs, compared to uninfected controls. Decreased mRNA levels of NEIL2, but not of OGG1, another oxidized DNA base repair enzyme as control, within the infected lung was further assessed by using real-time quantitative PCR (RT-qPCR) (**Supplementary Fig. 2c**). To further confirm these observations, immunoblots were performed for assessing the expression of several BER proteins in the nuclear extracts of uninfected and SARS-CoV-2 infected lungs of hamster at 10 dpi. Again, NEIL2 remains to be the only protein whose expression was significantly downregulated upon SARS-CoV-2 infection; in contrast to the expression of other BER proteins, such as OGG1, NEIL1 and AP-endonuclase1 (APE1), which were largely unchanged (**Supplementary Fig. 2d**). We reported earlier that the loss of NEIL2 leads to significant accumulation of oxidative DNA damage in the animal model and cultured cells (33-35), we thus analyzed DNA damage accumulation in the lungs of SARS-CoV-2-infected vs. uninfected hamsters using Long Amplicon-based qPCR (LA-qPCR) (39). Indeed, SARS-CoV-2-infected animals showed a significant increase in DNA damage accumulation (**Supplementary Fig. 2e**), consistent with the outcome of a decreased level of NEIL2 in those animals (33). Together, these data suggest a close link between decreased NEIL2 expression and the severe outcome of SARS-CoV-2 infection.

### Correlation of NEIL2 levels and prognosis of COVID-19 based on patient’s sex or age

**Figure 2:**
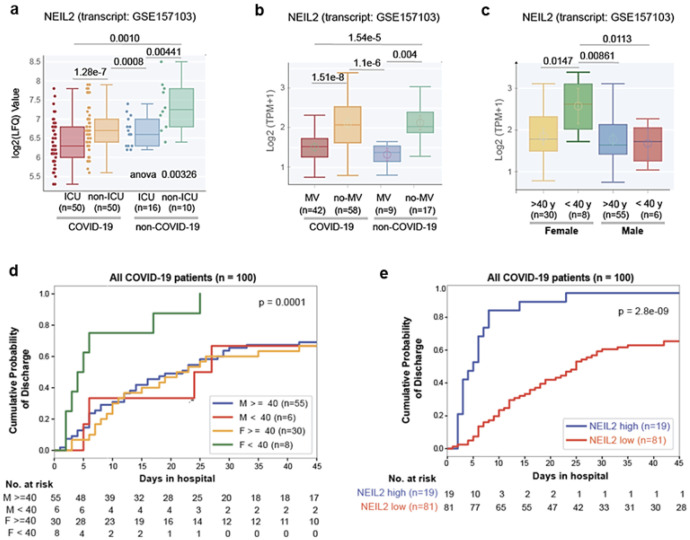
Correlation between NEIL2 levels and severity and prognosis of COVID-19 based on patient’s sex or age **a, b**) Whisker plots display the levels of expression of NEIL2 in a cohort of hospitalized patients (GSE157103), stratified based on their level of care (Intensive Care Unit [ICU] vs. non-ICU) (a) or requirement of Mechanical Ventilator (MV vs. no-MV) (b) and diagnosis (COVID-19 vs. non-COVID-19). **c**) Whisker plots display the levels of expression of NEIL2 in groups of patients (GSE157103) stratified by sex and age (using 40 years as a cut-off). **d, e**) Kaplan-Meier plots display the cumulative probability of discharge from the hospital stratified by sex and age (d) and high vs. low levels of NEIL2 expression (e).

Next, we investigated the prognostic potential of NEIL2 expression for COVID-19 severity, such as the need for ICU admission and the use of mechanical ventilator (MV), in COVID-19 and non-COVID-19 patient populations. Patients requiring ICU or MV had considerably lower levels of NEIL2 than non-ICU or non-MV patients (Fig. 2a,b); however, no such relationship was found for other DNA glycosylases, such as OGG1 and NEIL3 (**Supplementary Fig. 3a,b**). Moreover, among hospitalized COVID-19 patients, females aged 40 years or less had significantly higher NEIL2 levels (Fig. 2c) which coincided with their shorter duration of hospitalization compared to males in the same age group (Fig. 2d). These findings support the notion that the sex disparity in COVID-19-related severity/deaths puts males at a greater risk, and that this risk markedly increases with age in both sexes (40). In the case of OGG1/NEIL3, no such age/sex-specific trends were observed (**Supplementary Fig. 3c,d**). Furthermore, a study involving 100 hospitalized COVID-19 patients showed a significant correlation between higher NEIL2 levels and a shorter duration of hospitalization (Fig. 2e), unlike OGG1 and NEIL3 levels, which showed no correlation to hospital stay (**Supplementary Fig. 3e,f**). Collectively, these observations again support a strong link between NEIL2 deficiency and COVID-19 severity.

### Increased morbidity and mortality of *Neil2^−/−^* mice upon SARS-CoV-2/MA10 infection

**Figure 3:**
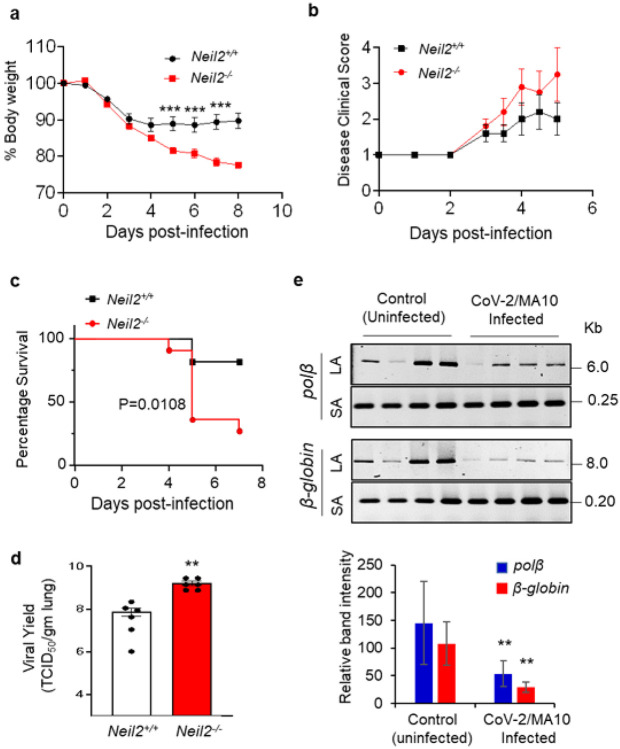
Increased mortality and DNA damage in the lungs of *Neil2^−/−^* vs. *Neil2^+/+^* mice following infection with CoV-2/MA10 **a, b**) *Neil2^−/−^* and *Neil2^+/+^* mice (n=10, each) were challenged intranasally with 1x10^5^ TCID_50_ of CoV-2/MA10 and monitored for 8 days for changes in % body weight (a) and disease clinical score/illness that was assessed based on a standardized 1 to 4 grading system (b); mean % body weight loss of *Neil2^−/−^* mice were significantly higher at 5, 6 and 7 dpi compared to *Neil2^+/+^*, *** p<0.005. **c**) *Neil2^−/−^* and *Neil2^+/+^* mice were monitored for 7 days following CoV-2/MA10 infection (1x10^5^ TCID_50_, intranasally) and the mortality of *Neil2^−/−^* vs. *Neil2^+/+^* mice are represented in a percent survival plot. **d**) *Neil2^−/−^* and *Neil2^+/+^* mice were challenged intranasally with 1x10^5^ TCID_50_ of CoV-2/MA10. Six mice from each group were euthanized at 2 dpi and lungs collected for viral titration. Lung viral yield was measured by standard TCID_50_ assay using VeroE6 cells and the viral yield/gram of lung weight was plotted at log10 scale. LOD: limit of detection 1.45x10^3^ TCID_50_/gm. **e**) Amplification of a large amplicon (6-8 kb) and a short amplicon (~200 bp) of the *Polβ* and *β-globin* genes from genomic DNA of uninfected and CoV-2/MA10 infected *Neil2^+/+^* mice lungs, at 5 dpi. Histogram shows the normalized relative band intensities (n=4). Error bars represent ± standard deviation from the mean. **=p <0.01 vs. *Neil2^+/+^* groups for d, and vs. uninfected controls for e.

The results presented above led us to hypothesize that low levels of NEIL2 play a critical role in an exacerbated outcome of SARS-CoV-2 infection. To test the biological significance of NEIL2 in COVID-19 pathogenesis, we utilized the *Neil2^−/−^* mouse model developed in our lab (33). *Neil2^−/−^* and *Neil2^+/+^* mice were infected with the mouse-adapted SARS-CoV-2-MA10 (CoV-2/MA10) that could productively infect mice resulting in weight loss and mortality in an age-dependent manner (41), providing an opportunity to explore the impact of NEIL2 on viral infection. Six to seven months old *Neil2^+/+^* and *Neil2^−/−^* mice were infected intranasally (i.n) with 1x10^5^ TICD_50_ of CoV-2/MA10 strain and were monitored daily for the onset of morbidity (i.e., weight changes and other signs of illness) and mortality (if any). Those animals reaching to the end-stage of clinical disease (>20% weight loss) were euthanized to assess the yields of infectious progeny virus within the lungs. We noted that infected *Neil2^−/−^* mice were losing weight more rapidly than the *Neil2^+/+^* cohort (Fig. 3a) concomitant with the onset of other signs of illness (Fig. 3b). More importantly, 80% of *Neil2^−/−^* mice succumbed to infection within 4-6 days compared to 20% mortality in *Neil2^+/+^* mice (Fig. 3c). Additionally, yields of infectious virus within the lung of *Neil2^−/−^* mice were significantly higher (~ 20-fold) than that of *Neil2^+/+^* mice at 2 dpi (Fig. 3d). Furthermore, a significant decrease in NEIL2, but not OGG1 or NEIL1, transcript was observed in the lungs of CoV-2/MA10 infected *Neil2^+/+^* mice (**Supplementary Fig. 4a-c**), which is consistent with our finding in SARS-CoV-2 infected permissive hamster model. Such a decrease in expression of NEIL2 at RNA level was subsequently confirmed by the immunoblotting which showed that the levels of NEIL2 protein in infected *Neil2^+/+^* mice were significantly decreased compared to uninfected controls (**Supplementary Fig. 4d**). As reduced NEIL2 expressions usually resulted in increased phosphorylated γH2AX (pSer139), a sensitive marker of double strand breaks (39), we investigated whether such a reverse correlation of the expression of NEIL2 and γH2AX could occur in SARS-CoV-2 infected mice. We indeed observed a significant increase in γH2AX (**Supplementary Fig. 4d**), thereby implying increased DNA double strand break accumulation in the lungs of CoV-2/MA10 infected mice. We validated the increased amount of DNA strand-break accumulation in CoV2/MA10 infected mouse lung using LA-qPCR analysis (Fig. 3e). Of note, we could not measure DNA damage in *Neil2^−/−^* mice because those (80%) died within 5 days. Collectively, these results suggest that excessive genome damage due to decreased NEIL2 level contributes to exacerbation of SARS-CoV-2 infection in COVID-19.

### CoV2/MA10-infection induced inflammatory responses in *Neil2^−/−^* mice

**Figure 4:**
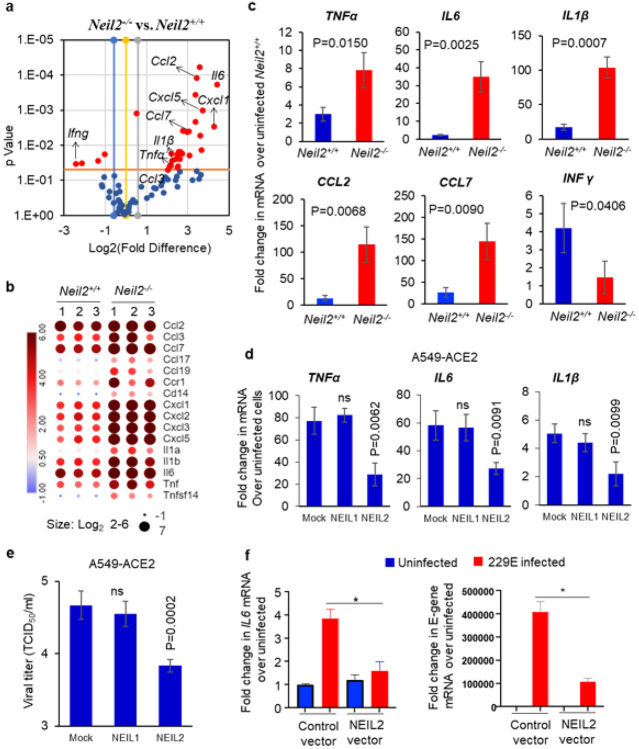
Inflammatory gene expression in the lungs of *Neil2^−/−^* vs. *Neil2^+/+^* mice following infection with CoV-2/MA10. **a**) Volcano plot of mRNA expression of proinflammatory genes in *Neil2^−/−^* vs. *Neil2^+/+^* mouse lung, 5 days post CoV-2/MA10 infection; x-axis, Log2-fold change; y-axis, p-value; the red dots depict differentially expressed genes with a p-value <0.05 and a fold change >1.5 between the two groups. **b**) The heatmap displays changes in the expression of a subset of proinflammatory genes associated with cytokine storm observed in COVID-19 patients, in *Neil2^−/−^* vs. *Neil2^+/+^* mice lungs, post CoV-2/MA10 infection. The color bar indicates the Log2-fold change in the transcript level; red and blue colors indicate high and low expression levels, respectively. **c**) Validation of multiplex array data for expression of indicated genes by RT-qPCR in CoV-2/MA10 infected *Neil2^+/+^* or *Neil2^−/−^* mice lung relative to uninfected *Neil2^+/+^* mice lung. Results are normalized to *18S*RNA. **d, e**) A549 cells expressing ACE2 (A549-ACE2) were transduced with mock (PBS+ carrier), rNEIL1 or rNEIL2 proteins for 48 h, then infected with SARS-CoV-2 (WA1-2020 at MOI 1). Total RNA was isolated for mRNA expression analysis using RT-qPCR (d) and supernatants harvested at 24 h post-infection for viral titer measurement using standard VeroE6 viral titration assay for the supernatants to determine TCID_50_/mL and plotted at Log10 scale (e). **f**) AGS cells transfected with control vector (control-vector) or NEIL2 expressing vector (NEIL2 vector) were infected with 229E strain and expression of host *IL6* (left panel) or viral E-gene (right panel) was analyzed using RT-qPCR compared to uninfected cells 72 h post infection. Results are normalized to *18S*RNA. Error bars represent ± standard deviation from the mean (n≥3); ns = not significant, *=p <0.05 vs. uninfected control vector expressing cells for f.

Given that NEIL2 is significantly downregulated in SARS-CoV-2 infected patients, and in both hamster and mouse models, we conducted a multiplex RT-qPCR analysis of 84 inflammation-associated genes in the lungs of CoV-2/MA10 infected *Neil2^−/−^* vs. *Neil2^+/+^* mice. As shown in Fig. 4a, infected *Neil2^−/−^* lungs had elevated (more than 2-fold) expression of ~47 genes and decreased expression of ~8 genes, compared to infected *Neil2^+/+^* lungs. Interestingly, a significant decrease was observed in the anti-viral cytokine IFNγ (p=0.0345). Fig. 4b shows differential expression of some of the critical cytokines in *Neil2^−/−^* vs. *Neil2^+/+^* mice that were found to be highly expressed in severe COVID-19 patients. The multiplex assay was further validated for a subset of significantly altered genes using RT-qPCR (Fig. 4c). Collectively, these data imply that NEIL2 deficiency or downregulation following viral infection plays a significant role in the host immune response and organ damage that are commonly linked with severe COVID-19.

Given that NEIL2 is an anti-inflammatory protein and in view of emerging success of protein therapy, we tested if exogenously added recombinant NEIL2 (rNEIL2) can limit CoV2-infection induced inflammatory responses. A549 cells expressing angiotensin converting enzyme-2 (ACE-2), the receptor for the SARS-CoV-2 viral entry (42), were transduced with rNEIL2, rNEIL1 or mock as control, and then infected with SARS-CoV-2 at the MOI of 1 for 24 h. *TNFa, IL6* and *IL1β* mRNA levels were all significantly reduced in rNEIL2 vs. mock transduced cells (Fig. 4d). In contrast, rNEIL1 transduced cells showed no significant change in mRNA levels compared to control cells. Surprisingly, we discovered that rNEIL2 transduced cells had fewer viral progeny (Fig. 4e) and lower viral E-gene expression (**Supplementary Fig. 5**), as compared to mock or rNEIL1 transduced cells. Similarly, overexpressing NEIL2 in human gastric adenocarcinoma, AGS cells infected with the human coronavirus 229E strain significantly decreased *IL6* transcript levels (Fig. 4f, left panel), and displayed lower levels of viral E-gene transcript (Fig. 4f, right panel), in comparison to infected control vector expressing cells. All these findings clearly suggest that NEIL2 plays a protective antiviral role against SARS-CoV-2 using multiple mechanisms.

### NEIL2 interacts with 5’-UTR of SARS-CoV-2 RNA

**Figure 5:**
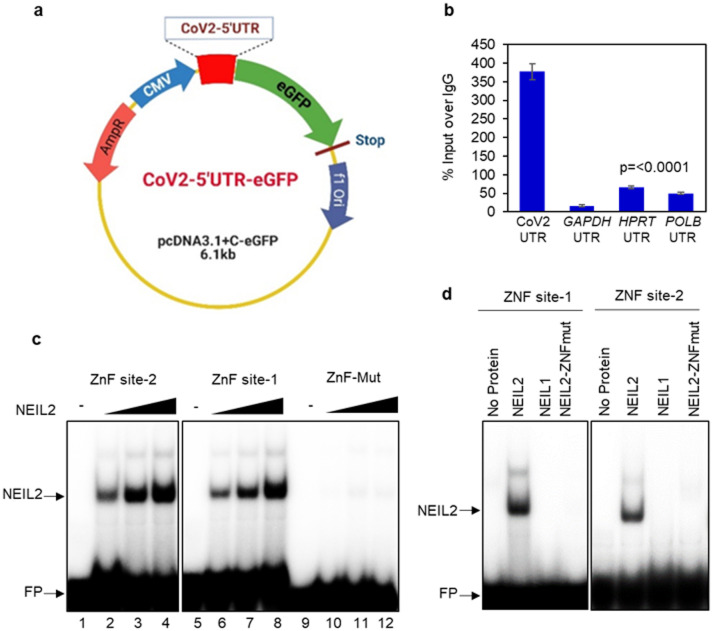
NEIL2 interacts with 5’-UTR of SARS-CoV-2 mRNA via ZnF motif. **a**) 5’-UTR of SARS-CoV-2 RNA (~300nt) was cloned upstream of Green Fluorescence Protein (eGFP) in mammalian expression plasmid under the control of CMV promoter as shown in the schematic. **b**) Human BEAS-2B cells stably expressing NEIL2-FLAG protein were transfected with CoV2-5’-UTR-eGFP construct and their nuclear extracts (NE) subjected to RNA chromatin immunoprecipitation analysis using the anti-FLAG or -IgG antibodies. RT-qPCR was carried out with 5’-UTR region specific primers for CoV2, *GAPDH, HPRT*, and *POLB* mRNAs. Results are represented as % inputs over IgG where error bars show ±standard deviation from the mean, n=3. **c**) RNA-Electrophoretic mobility-shift assay (RNA-EMSA) of binding of NEIL2 (25-100 ng) with ^32^P-labeled RNA probe containing zinc finger (ZnF) binding sites (site-1, lanes 6-8 and site-2, lanes 2-4) derived from SARS-CoV-2-5’-UTR or a RNA probe devoid of ZnF site (ZnF-mut, lanes 10-12). **d**) RNA-EMSA of binding of 50 ng NEIL2, NEIL1 or ZnF-mutant NEIL2 with ^32^P-labeled RNA probe containing ZnF-site-1 and ZnF-site-2 derived from SARS-CoV-2-5’-UTR. Representative images from 3 independent experiments are shown; FP represents free probe.

Like other b-CoVs, SARS-CoV-2 possesses a long RNA genome flanked by 5’- and 3’-translated regions (UTRs), containing regulatory cis-acting elements and very stable secondary RNA structure, essential for translation and RNA synthesis (43-45). Several host proteins interact with the 5’- and 3’-UTRs of viral RNA either to facilitate or hinder viral protein and RNA synthesis (43, 45-48). The suppression of viral progeny and E-gene expression in the presence of rNEIL2 prompted us to test whether NEIL2 is directly involved in the regulation of the viral life cycle via its interaction with CoV-2 RNA. We cloned the 5’-UTR of SARS-CoV-2 mRNA upstream of Green Fluorescence protein (eGFP) in the pcDNA3.1 vector (CoV2-5’-UTR-eGFP, Fig 5a) and transfected into human lung epithelial BEAS-2B cells, stably expressing FLAG-tagged NEIL2 (NEIL2-FLAG). Sixteen hours post transfection, the cell lysates were subjected to RNA chromatin immunoprecipitation (RNA-ChIP) using anti-FLAG antibody, followed by RT-qPCR analysis. Indeed, we detected strong association of NEIL2 with full length SARS-CoV-2 5’-UTR, but not with the 5’-UTR of several host genes; *GAPDH, HPRT* and DNA polymerase β (*POLB*) as controls in the RNA-ChIP analysis (Fig. 5b). Control reactions without reverse transcriptase ruled out the possibility of DNA contamination in the samples (**Supplementary Fig. 6a**).

The coronavirus RNA 5’-UTR and 3’-UTR contain cis-acting sequences that are functionally important for the binding of viral and host cellular proteins during translation and RNA replication (43, 49-51). Using RNA binding motif search tools (http://www.csbio.sjtu.edu.cn/bioinf/RBPsuite/, http://rbpmap.technion.ac.il/ and http://cisbp-rna.ccbr.utoronto.ca), we identified two zinc finger (ZnF) binding sites (site-1, nt 148-172 and site-2, nt 209-233, **Supplementary Table**) in the SARS-CoV-2 5’-UTR. RNA electrophoretic mobility shift assay (RNA-EMSA) showed robust sequence specific and dose-dependent binding of the NEIL2 to both of these sites containing RNA oligos (Fig. 5c, site-1, lanes 6-8; site-2, lanes 2-4), but not to the control or mutant RNAoligo (lanes 10-12). Inability of the binding of NEIL1 or of the ZnF mutant (C315S) NEIL2 to the 5’-UTR sites of CoV-2 underscored the specificity of NEIL2 RNA binding (Fig. 5d). We also found one putative ZnF binding site in the 3’-UTR (**Supplementary Table**), and recombinant NEIL2 showed a dose dependent binding to 3’-UTR-ZnF site of the SARS-CoV-2 RNA oligo, as analyzed by RNA-EMSA (**Supplementary Fig. 6b**). To assess whether such binding has any effect on SARS-CoV-2 replication, we examined *in vitro* viral RNA dependent RNA polymerase (RdRp, nonstructural protein, nsp12 in complex with accessory, nsp7 and nsp8, **Supplementary Fig. 6c**) activity using independent RNA oligos containing SARS-CoV-2 5’-UTR-ZnF sites or SARS-CoV-2 3’-UTR-ZnF site sequences as template RNA and short complementary oligo sequences as primers in the presence or absence of rNEIL2 to initiate 5’-3’ extension. However, we did not detect any inhibition in viral RdRp activity *in vitro* (**Supplementary Fig. 6d,e**). We thus, postulated that NEIL2 regulates viral protein synthesis by blocking activity of host translational machinery at the 5’-UTR of CoV-2.

### NEIL2 suppresses SARS-CoV-2-5’-UTR-mediated protein expression

**Figure 6:**
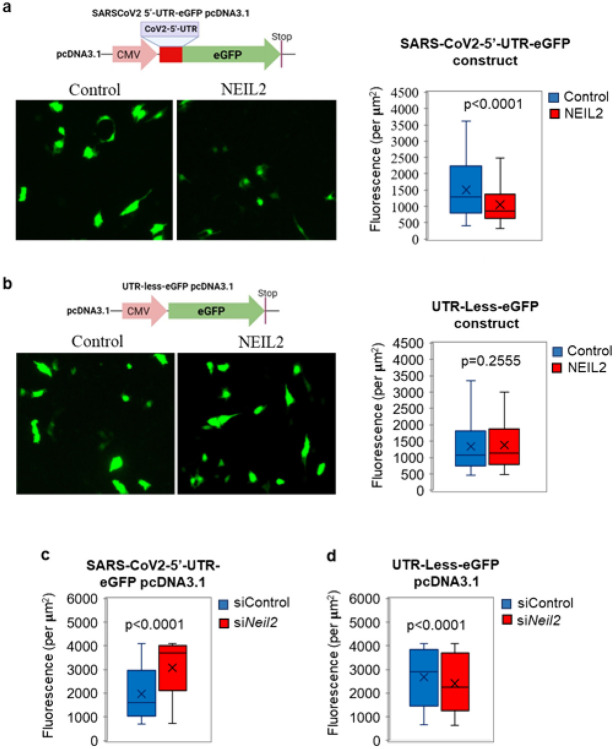
NEIL2 modulates SARS-CoV-2 protein synthesis. **a, b**) Human BEAS-2B cells, control or stably expressing NEIL2-FLAG protein (NEIL2) were transfected with CoV2-5’-UTR-eGFP construct (a) or a plasmid devoid of CoV-2-5’-UTR (UTR-Less-eGFP) (b) and GFP protein expression was analyzed in live cells after 16 h. Representative images from at least 10 randomly selected frames (left panels). Right panels represents GFP Fluorescence per μM^2^ from at least 10 randomly selected frames. **c, d**) HEK293 cells were transfected with Control or -NEIL2 specific siRNA for 48 h, then transfected with CoV2-5’-UTR-eGFP construct (c) or UTR-Less-eGFP plasmid (d), and GFP fluorescence per μM^2^ was measured in live cells from at least 10 randomly selected frames, p-values calculated from at least three biological replicates.

To examine the regulatory function of NEIL2 in viral protein expression, we transfected SARS-CoV-2-5’-UTR-eGFP plasmid (Fig. 4a) or UTR-Less-eGFP plasmid into NEIL2-FLAG overexpressing or control BEAS-2B cells; and GFP fluorescence was analyzed as a measure of expression of the protein, 12-16 h post transfection. Both SARS-CoV-2-5’-UTR-eGFP and UTR-less-eGFP plasmid transfected cells showed comparable GFP DNA (as a measure of transfection efficiency) in NEIL2 overexpressing vs. control cells as analyzed by qPCR (**Supplementary Fig. 7a**). Intriguingly, we observed that the GFP expression was significantly decreased at the protein level (Fig. 6a), but not at the transcript level (**Supplementary Fig. 7b**) in NEIL2 overexpressing cells compared to control cells, when transfected with the SARS-CoV-2-5’-UTR-eGFP construct. However, no significant change in GFP expression was observed between control or NEIL2 overexpressing cells transfected with UTR-Less-eGFP construct (Fig. 6b). Furthermore, siRNA mediated NEIL2 depletion (**Supplementary Fig. 7c**) in HEK-293 cells resulted in significantly higher expression of GFP in cells transfected with SARS-CoV-2-5’-UTR-eGFP construct compared to control siRNA-treated (siControl) cells (Fig. 6c), while the UTR-Less-eGFP plasmid transfected into NEIL2-deficient (si*Neil2*) cells showed only a modest decrease in GFP expression (Fig. 6d). Collectively, these data suggest that NEIL2 binds to 5’-UTR of CoV-2 RNA and blocks the translational machinery and thus, decreased GFP expression. However, in the absence of NEIL2, the 5’-UTR was readily accessible to the host protein synthesis machinery resulting in increased GFP expression. All these data strongly suggest a role of NEIL2 in inhibition of SARS-CoV-2 protein synthesis.

## Discussion

Dysregulation and often exacerbation of immune responses caused by viral infections could result in severe tissue damage, eventually leading to multiorgan failure and death. The excessive reactive oxygen species generated as a result cause damage to genomic DNA and activate DNA damage response (DDR) pathways (52, 53). We thus postulated that individuals with compromised DNA repair capacity would be more prone to severe CoV-2 infection. However, to date, there is no report describing the linkage between SARS-CoV-2 infection and host genome damage-induced signaling, or the role of DNA repair proteins therein. Here we report that the level of the DNA glycosylase, NEIL2, is significantly low at both transcript and protein levels in severe COVID-19 patients. We further investigated the role of NEIL2 in SARS-CoV-2 infection and pathogenicity using permissive animal models. Using the mouse adaptive strain of SARS-CoV-2, CoV-2/MA10, which captures various aspects of severe COVID-19 disease such as elevated cytokines, the loss of pulmonary function linked to ARDS, and the spectrum of morbidity and mortality of COVID-19 disease in an age dependent manner (41), we found that CoV-2/MA10 infected *Neil2^−/−^* mice had higher viral load, significant weight loss and mortality within 6–7 days. CoV-2/MA10 infection also significantly decreased NEIL2 levels with a concomitant increase in DNA damage in the lungs of wild type mice compared to uninfected animals. SARS-CoV-2 infected golden Syrian hamsters also showed decreased expression of NEIL2 and higher DNA damage. These observations are in accordance with earlier studies that *Neil2^−/−^* mice accumulate significant amount of DNA damage (33).

In addition to the canonical function of repairing genome damage, the work presented here also elucidated two non-canonical functions of NEIL2 that can explain its protective role against SARS-CoV-2 infection. We recently reported that NEIL2 acts as a repressor of NF-κB, a transcriptional activator of proinflammatory genes (36). We have demonstrated that expression of inflammatory genes is significantly higher in *Neil2^−/−^* mice; however, intrapulmonary delivery of rNEIL2 prevented TNFα-induced NF-κB recruitment to the promoters of cytokine genes both *in vitro* and *in vivo* (36). Similarly, we found that transduction of rNEIL2 in A549-ACE2 cells significantly inhibited SARS-CoV-2 induced *TNFα, IL6* and *IL1β* expression, further confirming anti-inflammatory role of NEIL2. Therefore, NEIL2 is able to mitigate the viral-induced ‘cytokine storm’ in the host by acting as a repressor of proinflammatory gene expression.

The success of viruses as pathogens depends on their ability to actively reprogram the host cell antiviral defense mechanisms. Activation of antiviral innate immune signaling cascade generally begins with recognition of viral genomes by intracellular pattern recognition receptors (54, 55) or by a set of zinc finger proteins (ZFPs), such as zinc-finger antiviral proteins (ZAP and PARP13), monocyte chemoattractant protein 1-induced protein 1 (MCPIP1) and ZCCHC7 that detect viral RNAs and elicit subsequent antiviral responses. In most known cases, these ZFPs recruit both the 5'- and 3'- mRNA decay machinery to degrade the target RNAs (56-60). Suppression of viral replication can occur via translational repression of its own proteins, as has been shown for influenza A virus NS1 mRNA by zinc finger protein 36 (58). ZAP also has an inhibitory effect on the viral translation by disrupting the interaction between eIF4G and eIF4A (61). Similarly, we provide evidence here that NEIL2, a ZFP (62), also directly interacts with the CoV-2 5’-UTR and blocks protein synthesis. This data is in accordance with previous reports showing that host protein impedes viral protein synthesis via binding to 5’- or 3’-UTRs of viral RNA (58, 61). The translational initiation for SARS-CoV-2 RNA is not completely understood. Some reports suggest that the translation mechanism of SARS-CoV-2 RNA is independent of cap-binding translation factors (eIF4E and eIF4F) (43). Also, the CoV-2-5’-UTR is rich in GC content and is capable of forming internal ribosome entry sites to recruit host ribosomes for translating its RNA (63). We postulate that NEIL2 binding to the 5’-UTR will inhibit ribosome entry or interfere with the assembly of translational machinery and inhibit viral protein synthesis, which warrants further investigation in the future.

The interplay between the virus and many host factors plays critical roles in determining the final outcomes of viral infection. Here, we show that NEIL2 is an important host factor for providing protection against SARS-CoV-2 infection. Based on the work presented here, we propose that if the levels of NEIL2 in hosts are low, or the virus is able to significantly diminish the levels of NEIL2, there is a greater chance for successful viral life cycle. In support of this notion, we found a correlation between NEIL2 levels and differences in age or sex associated with the risk of severe COVID-19. Additionally, lower levels of NEIL2 not only correlated with a longer period of hospitalization but also with higher instances of admission to ICU or the requirement of MVs among patients with severe disease. Moreover, several studies pointed towards the activation of NF-κB-mediated inflammation to explain the importance of age and sex in COVID-19 severity (40, 64-66). The NF-κB pathway induces a pro-inflammatory phenotype, known as inflamm-aging, in older patients that is associated with increased levels of oxidative stress, thus driving the sustained levels of inflammation and DNA damage leading to cellular DDR, and further expression of *IL-6* (64, 65). It is imperative that NEIL2 coordinates with other host factors to mount a defense against viral infection. Future experiments are required to delineate the coordination between NEIL2 and different host factors. Collectively, here we demonstrate multi-faceted functions of the host DNA repair enzyme NEIL2, where it unconventionally regulates COVID-19 pathogenesis, by decreasing host inflammatory response, inhibiting CoV-2 replication, and repairing host genome damage, thereby mitigating disease severity. Finally, the ability of rNEIL2 to neutralize the effect of SARS-CoV-2 in cultured cells, suggests that it has a strong therapeutic potential as a biologic against COVID-19.

## Methods

### Analysis of RNASeq Datasets

Publicly available COVID-19 gene expression databases were downloaded from the National Center for Biotechnology Information (NCBI) Gene Expression Omnibus website (GEO) (67-69). If the dataset was not normalized, RMA (Robust Multichip Average) (70,71) was used for microarrays and TPM (Transcripts Per Millions) (72,73) was used for RNASeq data for normalization. We used log2 (TPM+1) to compute the final log-reduced expression values for RNASeq data. Accession numbers for these crowd sourced datasets are provided in the figures and manuscript. Single Cell RNASeq data from GSE145926 was downloaded from GEO in the HDF5 Feature Barcode Matrix Format. The filtered barcode data matrix was processed using Seurat v3 R package (74). Pseudo bulk analysis of GSE145926 data was performed by adding counts from the different cell subtypes and normalized using log2 (CPM+1). All of the above datasets were processed using the Hegemon data analysis framework (75-77).

### Immunohistochemistry (IHC)

COVID-19 samples were inactivated by storing in 10% formalin for 2 days and then transferred to zinc-formalin solution for another 3 days. The deactivated tissues were transferred to 70% ethanol and cassettes were prepared for tissue sectioning. The slides containing hamster and human lung tissue sections were de-paraffinized in xylene (Sigma-Aldrich, catalog no. 534056) and rehydrated in graded alcohols to water. For NEIL2 antigen retrieval, slides were immersed in Tris-EDTA buffer (pH 9.0) and boiled for 10 minutes at 100°C inside a pressure cooker. Endogenous peroxidase activity was blocked by incubation with 3% H_2_O_2_ for 10 minutes. To block non-specific protein binding 2.5% goat serum (Vector Laboratories, catalog no. MP-7401) was added. Tissues were then incubated with rabbit anti-NEIL2 polyclonal antibody (in house, 62) for 1.5 h at room temperature in a humidified chamber and then rinsed with TBS or PBS 3x, 5 minutes each. Sections were incubated with horse anti-rabbit IgG (Vector Laboratories, catalog no. MP-7401) secondary antibodies for 30 minutes at room temperature and then washed with TBS or PBS 3x, 5 minutes each; incubated with DAB (3,3'-diaminobenzidine tetrahydrochloride) (Thermo Scientific, catalog no. 34002), counterstained with hematoxylin (Sigma-Aldrich, catalog no. MHS1) for 30 seconds, dehydrated in graded alcohols, cleared in xylene, and cover slipped. Epithelial and stromal components of the lung tissue were identified by staining duplicate slides in parallel with hematoxylin and eosin (Sigma-Aldrich, catalog no. E4009) and visualizing by Leica DM1000 LED (Leica Microsystems, Germany).

### IHC Quantification

IHC images were randomly sampled at different 300x300 pixel regions of interest (ROI). The ROIs were analyzed using IHC Profiler (78). IHC Profiler uses a spectral deconvolution method of DAB/hematoxylin color spectra by using optimized optical density vectors of the color deconvolution plugin for proper separation of the DAB color spectra. The histogram of the DAB intensity was divided into 4 zones: high positive (0 to 60), positive (61 to 120), low positive (121 to 180) and negative (181 to 235). High positive, positive, and low positive percentages were combined to compute the final percentage positive for each ROI. The range of values for the percent positive is compared among different experimental groups.

### Lung tissue specimens from the rapid autopsy procedure

Lung specimens from COVID-19 positive human subjects were collected using autopsy procedures at the University of California San Diego (the study was IRB Exempt) following guidelines from the CDC and CAP autopsy committee. All donations to this trial were obtained after telephone consent followed by written email confirmation with next of kin/power of attorney per California state law (no in-person visitation could be allowed into the COVID-19 ICU during the pandemic). (https://www.cdc.gov/coronavirus/2019-ncov/hcp/guidance-postmortem-specimens.html and https://documents.cap.org/documents/COVID-Autopsy-Statement-05may2020.pdf). Lung specimens were collected in 10 % Zinc-formalin and stored for 72 h before processing for histology as done previously (79,80).

### Animals

Lung samples were collected from 8 week-old Syrian hamsters post SARS-CoV-2 infection conducted exactly as in a previously published study (38). Briefly, lungs from hamsters challenged with SARS-CoV-2 (1 × 10^6^ PFU) were harvested on day 5 (peak weight loss) and NEIL2 protein and mRNA levels were analyzed by IHC and quantitative Polymerase Chain Reaction (qPCR), respectively. Nuclear extract was prepared from the uninfected and infected hamsters lungs at 10 days post infection, and DNA was extracted from the same samples for Long Amplicon qPCR (LA-qPCR). The generation of *Neil2^−/−^* mice (C57BL/6J congenic) background was described previously (33). The research protocol was approved and performed in accordance with Scripps Research Institutional Animal Care and Use Committee (IACUC) protocol no. 20-0003, (Hazra, protocol no. 0606029D, and Boldogh, protocol no. 0807044D).

SARS-CoV-2-MA10 infection studies in 6 month-old *Neil2^+/+^* and *Neil2^−/−^* mice were carried out at Galveston National Laboratory at UTMB, an AAALAC accredited (November 24, 2020) and PHS OLAW approved (February 26, 2021) high-containment National Laboratory, based on a protocol approved by the Institutional Animal Care and Use Committee at UTMB at Galveston (Tseng protocol no. 2004052). Six-month-old, *Neil2^+/+^* and *Neil2^−/−^* (16 each) mice were challenged with 1x10^5^ TCID_50_ mouse adapted strain of SARS-CoV-2 MA10 (CoV2/MA10) and observed daily for body weight change, mortality and clinical score/wellbeing. Clinical wellbeing of mice was scored based on a 1–4 standardized grading system. Score 1 is healthy; score 2 is with ruffled fur and lethargic; score 3 is with additional clinical sign such as hunched posture, orbital tightening, increased respiratory rate, and/or > 15% weight loss; score 4 is showing dyspnea, reluctance to move when stimulated, or ≥ 20% weight loss that needs immediate euthanasia. Six mice from each group were euthanized at 2 day post-infection to assess the lung viral load by TCID_50_.

### Cell culture and Transient transfection

Human bronchial epithelium cell line, BEAS2B (ATCC® CRL-9609™) stably expressing NEIL2-FLAG and human embryonic kidney cells (HEK293; 81) were grown at 37°C and 5 % CO2 in DMEM/F-12 (1:1) containing 10 % fetal bovine serum, 100 units/ml penicillin and 100 units/ml streptomycin. For all experiments, 50-60 % confluent cells were used. We routinely test mycoplasma contaminations in all our cell lines using the PCR-based Venor™ GeM Mycoplasma Detection Kit (Sigma, catalog no. MP0025). Control or stable BEAS-2B cells at approximately 70 % confluency were transiently transfected with vector expressing GFP with (SARS-CoV2-5’-UTR-eGFP construct) or without (UTR-Less-eGFP construct) UTR (100 ng) using Lipofectamine TM 2000, according to the supplier’s protocol. To monitor transfection efficiency, a reporter gene construct (0.25 μg) containing β-galactosidase downstream to the SV40 promoter was co-transfected. Cells were allowed to recover for 16 h in media with serum and then GFP florescence was measured using an ECHO florescent microscope. Total RNA and DNA were isolated for subsequent qPCR analysis.

### Gene expression with real time-qPCR (RT-qPCR)

Total RNA extraction was performed from cells using TRIzol™ Reagent (Invitrogen™, catalog no. 15596026). Genomic DNA was removed and up to 2 μg RNA was used to synthesize cDNA with a PrimeScriptTM RT Kit with gDNA Eraser (TaKaRa, catalog no. RR047A). qPCR was carried out using TB Green™ Premix Ex Taq™ II (Tli RNase H Plus; TaKaRa, catalog no. RR820A) in Applied Biosystems™ 7500 Real-Time PCR Systems with thermal cycling conditions of 94°C for 5 min, (94°C for 10 s, and 60°C for 1 min) for 40 cycles, and 60°C for 5 min. The target mRNA levels were normalized to that of *Gapdh*. In each case, DNase-treated RNA samples without reverse transcriptase were used to rule out genomic DNA contamination.

### RNA Chromatin immunoprecipitation and quantitative PCR (RNA-ChIP)

RNA-ChIP assays were performed as described earlier (39). Briefly, cells were cross-linked in 1% formaldehyde for 10 min at room temperature. Then 125 mM Glycine was added to stop crosslinking and samples were incubated for 5 min at room temperature. Samples then were centrifuged at 1000 xg at 4°C for 5 min to pellet the cells. The cells were incubated in buffer A (5 mM HEPES, 85 mM KCl, 0.5% NP-40 and 1X Protease inhibitor cocktail (Roche, catalog no. 4693132001)) for 10 min at 4°C, then washed once with buffer B (buffer A minus NP-40) at 2,500 xg for 5 min to pellet the nucleus. The nuclear pellet was re-suspended in sonication buffer containing 50 mM Tris-HCl pH 8.0, 10 mM EDTA and 1% SDS with 1X Protease inhibitor cocktail and sonicated to an average DNA size of ~300 bp using a sonicator (Qsonica Sonicators). The supernatants were diluted with 15 mM Tris-HCl pH 8.0, 1.0 mM EDTA, 150 mM NaCl, 1% Triton X-100, 0.01% SDS and protease inhibitors, and incubated with ChIP grade anti- FLAG (Millipore, catalog no. F1804) or normal IgG (Santa Cruz, sc-2025) antibodies overnight at 4°C. Immunocomplexes (ICs) were captured by Protein A/G PLUS agarose beads (Santa Cruz, catalog no. sc-2003) that were then washed sequentially in buffer I (20 mM Tris-HCl pH 8.0, 150 mM NaCl, 1 mM EDTA, 1% Triton-X-100 and 0.1% SDS); buffer II (same as buffer I, except containing 500 mM NaCl); buffer III (1% NP-40, 1% sodium deoxycholate, 10 mM Tris-HCl pH 8.0, 1 mM EDTA); and finally with 1X Tris-EDTA (pH 8.0) buffer at 4°C for 5 min each. 50 U ml^−1^ of RNase inhibitor (Roche, catalog no. 03335402001) was added to buffers A and B, sonication and IP buffers, and 40 U ml^−1^ to each wash buffer. The ICs were extracted from the beads with elution buffer (1% SDS and 100 mM NaHCO_3_) and de-crosslinked for 2 h at 65°C. RNA isolation was carried out in acidic phenol–chloroform followed by ethanol precipitation with GlycoBlue (Life Technologies, catalog no. AM9516) as a carrier. Genomic DNA was removed and reverse transcription was performed using a PrimeScript RT Kit with gDNA Eraser (TaKaRa, catlog no. RR047A). RNA-ChIP samples were analyzed by qPCR using specific primers. qPCR data are represented as percentage input after normalization to IgG.

### Protein expression and purification

Wild-type recombinant His-tagged -NEIL2, -NEIL2-ZnF mutant and -NEIL1 proteins were purified from *E. coli* using protocol as described earlier (62). Briefly, pET22b (Novagen) vector containing C-terminal His tagged-protein Coding DNA Sequence (CDS) was transformed into *E.coli BL21 (DE3)* RIPL Codon-plus cells. The log-phase culture (A_600_ = 0.4-0.6) of *E. coli* was induced with 0.5 mM isopropyl-1-thio-β-D-galactopyranoside (IPTG) and grown at 16°C for 16 h. After centrifugation, the cell pellets were suspended in a lysis buffer (Buffer A) containing 25 mM Tris-HCl, pH 7.5, 500 mM NaCl, 10% glycerol, 1 mM ß-mercaptoethanol (ß-ME), 0.25% Tween 20, 5 mM imidazole, 2 mM phenylmethylsulfonyl fluoride (PMSF). After sonication, the lysates were spun down at 13,000 rpm and the supernatant was loaded onto HisPur™ Cobalt Superflow Agarose (Thermo Scientific™, catalog no. 25228) previously equilibrated with Buffer A and incubated for 2 h at 4°C. After washing with Buffer A with a gradient of increasing concentration of imidazole (10, 20, 30, 40 mM), the His-tagged proteins were eluted with an imidazole gradient (80-500 mM imidazole in buffer containing 25 mM Tris-HCl, pH-7.5, 300 mM NaCl, 10% glycerol, 1 mM ß-ME, 0.25% Tween 20). After elution, the peak protein fractions were dialyzed against Buffer C (1X PBS, pH 7.5, 1 mM dithiothreitol (DTT), and 25 % glycerol) and stored at −20°C in aliquots.

The novel Corona virus nsp12 (GenBank: MN908947) gene, cloned into a modified pET 24b vector, with the C-terminus possessing a 10 × His-tag, was a gift from Dr. Whitney Yin. The plasmid was transformed into *E. coli* BL21 (DE3), and the transformed cells were cultured at 37 °C in LB media containing 100 mg/L ampicillin. After the OD_600_ reached 0.8, the culture was cooled to 16 °C and supplemented with 0.5 mM IPTG. After overnight induction, the cells were harvested through centrifugation, and the pellets were re-suspended in lysis buffer (20 mM Tris-HCl, pH 8.0, 150 mM NaCl, 4 mM MgCl2, 10% glycerol). The rest of the procedure is same as above with following modifications: the His-tagged protein was eluted with an imidazole gradient (80-250 mM imidazole in buffer containing 20 mM Tris-HCl, pH 8.0, 150 mM NaCl, 4 mM MgCl_2_, 10 % glycerol). Similarly, nsp7 and nsp8 genes, individually cloned in pET22b and pET30a+ vectors, respectively, were expressed in *E. coli* as described in case of NEIL proteins. After elution, the peak protein fractions of these proteins were dialyzed against Buffer D (20 mM Tris-HCl, pH 8.0, 250 mM NaCl, 1 mM DTT, 25% glycerol) and stored at −20°C in aliquots.

### Viral replication (RdRp) assay

For assembling the stable nsp12-nsp7-nsp8 complex, purified nsp12 was incubated with nsp7 and nsp8 at 4 °C for three hours, at a molar ratio of 1: 2: 2 in a buffer containing 20 mM Tris-HCl, pH 7.5, 250 mM NaCl and 4 mM MgCl_2_ (82).

For the RdRp assay, the 5’-monophosphorylated RNA templates (the portion of the template which is complementary to the 4-nt primer is underlined, **Supplementary Table**) were mixed at the following final concentrations in 20 μL reaction volume: Tris-HCl (pH 8, 25 mM), RNA primer (200 μM), RNA template (2 μM), [α^32^P]-UTP (0.1 μM), BSA (1 mg/ml), 0.1 μM GTP, CTP, ATP and 0.01 μM UTP and SARS-CoV-2 RdRp complex (~0.1 μM) on ice. For NEIL2 binding, the indicated concentrations of NEIL2 were incubated in the buffer with RNA on ice for 15 minutes. Reactions were stopped after 15, 30 or 60 min by the addition of 20 μL of a formamide/EDTA (50 mM) mixture and incubated at 95°C for 10 min. Data was collected and analyzed using a Typhoon FLA 7000 phosphorimager (GE Healthcare).

### RNA-Electrophoretic mobility-shift assay (RNA-EMSA)

Sequences of the oligonucleotide (oligo) probes used for RNA-EMSAs are listed in **Supplementary Fig 3D**. RNA-EMSA was performed as described before (83), with some modifications. Briefly, [α-^32^P]ATP labelled RNA oligo probes were incubated with 150-300 ng of purified protein in a binding buffer containing 10 mM Tris-Cl buffer (pH 7.6), 15 mM KCl, 5 mM MgCl_2_, 0.1 mM DTT, 10 units of RNase inhibitor, 1 mg BSA, and 0.2 mg/ml yeast tRNA in a 10 μl reaction volume. After a 15-min incubation on ice, 100 mg/ml of heparin was added and incubated for 10 min. RNA-protein complexes were resolved on a 5 % non-denaturing polyacrylamide gel at 120 V using 0.5x Tris-borate-EDTA as the running buffer at 4°C. Gels were fixed in an Acetone: Methanol: H_2_O (10:50:40) solution for 10 min, exposed to a Phosphor screen for 12-16 h and scanned using Typhoon FLA 7000 phosphorimager.

### Long Amplicon qPCR (LA-qPCR) assay

Lung tissues from freshly euthanized uninfected and SARS-CoV-2 infected hamsters and mice were used for DNA damage analysis. Genomic DNA was extracted using the Genomic tip 20/G kit (Qiagen) per the manufacturer’s protocol, to ensure minimal DNA oxidation during the isolation steps. The DNA was quantitated by Pico Green (Molecular Probes) in a black-bottomed 96-well plate and gene-specific LA qPCR assays were performed as described earlier (39) using Long Amp Taq DNA Polymerase (New England BioLabs). The LA-qPCR reaction was set for all genes from the same stock of diluted genomic DNA sample, to avoid variations in PCR amplification during sample preparation. Preliminary optimization of the assays was performed to ensure the linearity of PCR amplification with respect to the number of cycles and DNA concentration (10-15 ng). The final PCR reaction conditions were optimized at 94°C for 30 s; (94°C for 30 s, 55-60°C for 30 s depending on the oligo annealing temperature, 65°C for 10 min) for 25 cycles; 65°C for 10 min. Since amplification of a small region is independent of DNA damage, a small DNA fragment (~200-500 bp) from the corresponding gene(s) was also amplified for normalization of amplification of the large fragment. The amplified products were then visualized on gels and quantitated with ImageJ software (NIH). The extent of damage was calculated in terms of relative band intensity with the uninfected control mice/hamster sample considered as 100.

### Statistical analysis

All statistical tests were performed using R version 3.2.3 (2015-12-10). Standard t-tests were performed using python scipy.stats.ttest_ind package (version 0.19.0) with Welch’s Two Sample t-test (unpaired, unequal variance (equal_var=False), and unequal sample size) parameters. Multiple hypothesis correction was performed by adjusting p values with statsmodels.stats.multitest.multipletests (fdr_bh: Benjamini/Hochberg principles). The results were independently validated with R statistical software (R version 3.6.1; 2019-07-05). Pathway analysis of gene lists were carried out via the Reactome database and algorithm. Reactome identifies signaling and metabolic molecules and organizes their relations into biological pathways and processes. Kaplan-Meier analysis was performed using lifelines python package version 0.22.8. Violin and Swarm plots were created using python seaborn package version 0.10.1.

Two-sided unpaired Student’s t-test (http://www.ruf.rice.edu/~bioslabs/tools/stats/ttest.html) and online MedCalc statistical software (https://www.medcalc.org/calc/comparison_of_means.php) were used for analysis of statistical significance between two sets of data. The number of independent experiments denotes the number of biological replicates. Significance was evaluated at level P>0.05 (not significant), P<0.05 (*), P<0.01 (**) and P<0.005 (***), as the case may be.

## Data Availability

The data that support the findings of this study are available from the corresponding author upon request.
